# Contextual Regulation of TGF-β Signaling in Liver Cancer

**DOI:** 10.3390/cells8101235

**Published:** 2019-10-11

**Authors:** Shuo Tu, Wei Huang, Chunhong Huang, Zhijun Luo, Xiaohua Yan

**Affiliations:** 1 Department of Biochemistry and Molecular Biology, School of Basic Medical Sciences, Nanchang University, Nanchang 330006, China; tushuo@126.com (S.T.); HW18279561717@163.com (W.H.); chhuang@ncu.edu.cn (C.H.); 2 Department of Pathology, School of Basic Medical Sciences, Nanchang University, Nanchang 330006, China; zluo559914@ncu.edu.cn; 3 Institute of Biomedical Sciences, Nanchang University, Nanchang 330006, China

**Keywords:** TGF-β signaling, Smad, contextual regulation, liver cancer, hepatocellular carcinoma, cytostasis

## Abstract

Primary liver cancer is one of the leading causes for cancer-related death worldwide. Transforming growth factor beta (TGF-β) is a pleiotropic cytokine that signals through membrane receptors and intracellular Smad proteins, which enter the nucleus upon receptor activation and act as transcription factors. TGF-β inhibits liver tumorigenesis in the early stage by inducing cytostasis and apoptosis, but promotes malignant progression in more advanced stages by enhancing cancer cell survival, EMT, migration, invasion and finally metastasis. Understanding the molecular mechanisms underpinning the multi-faceted roles of TGF-β in liver cancer has become a persistent pursuit during the last two decades. Contextual regulation fine-tunes the robustness, duration and plasticity of TGF-β signaling, yielding versatile albeit specific responses. This involves multiple feedback and feed-forward regulatory loops and also the interplay between Smad signaling and non-Smad pathways. This review summarizes the known regulatory mechanisms of TGF-β signaling in liver cancer, and how they channel, skew and even switch the actions of TGF-β during cancer progression.

## 1. Introduction

Primary liver cancer is one of the most diagnosed cancer types and the third cause for cancer-related death worldwide, with approximately 841,000 new cases and 782,000 deaths each year [1,2]. As the major primary liver cancer type, hepatocellular carcinoma (HCC) alone accounts for about 75–85% of all incident cases [3,4]. Thereafter, liver cancer in this review mainly refers to HCC. HCC is originated from neoplastic hepatocytes, and usually develops following a long period of chronic liver diseases including hepatitis, fibrosis and cirrhosis [3,5,6]. The main risk factors for liver cancer include virus infection like HBV and HCV, alcohol abuse, nonalcoholic fatty liver disease (NAFLD), aflatoxin-contaminated foods, among others [1,3,4].

Liver cancer development is controlled by both extracellular factors and intracellular signaling pathways [5,7,8,9,10]. Compared with those in homeostatic liver, these pathways are significantly altered and rewired to favor tumorigenesis and cancer progression. Transforming growth factor beta (TGF-β) acts as a cytostatic factor in normal hepatocytes and most early-stage liver cancer cells [10,11,12]. Gene-targeting studies of TGF-β signaling components have verified this. Haploid deficiency of the TGF-β1 gene facilitates chemical-induced liver cancer [13], and TβRII-deficient mice exhibit higher tumor susceptibility [14]. In line with this, transgenic Smad3 in mice inhibits liver tumorigenesis by promoting TGF-β-induced apoptosis [15]. Intriguingly, TGF-β is unique in that it could be averted from a tumor suppressor to a promoter in liver cancer, by facilitating cancer cell proliferation, EMT, invasion and metastasis, in addition to modifying the tumor microenvironment [9,10,16,17]. In support of this, TGF-β has been found to be overexpressed in metastatic HCC tissues when compared with non-metastatic tissues or normal tissues [18,19,20,21]. Forced expression of TGF-β in mice, especially in the double transgenic mice with c-Myc or CyclinD1 expression, is able to promote liver tumorigenesis [22,23].

A striking nature of TGF-β is its multi-faceted roles and even opposite functions in different contexts or in distinct stages of cancer [24,25,26,27,28]. Some important progress has been achieved in understanding the underlying molecular bases. First of all, high-throughput studies have clearly established that context- or cell type-specific transcription factors play a crucial role in determining the genomic binding sites of Smad proteins, the major downstream signal transducers of TGF-β, thereby yielding contextual target gene expression patterns [25,26]. Second, TGF-β could activate some non-Smad signaling molecules including PI3K/Akt, MAPKs, PAK2, small Rho GTPases and others, relying on cell types and contexts [29]. These non-Smad molecules contribute to specific TGF-β responses via different mechanisms [10,12,30]. Third, although the simplified and linear TGF-β/Smad pathway is believed to fully recapitulate the context-dependent actions of TGF-β, the signaling intensity and duration are also critical in determining the outputs of TGF-β [10,25,26,30]. Various regulators and mechanisms have been discovered to control TGF-β signaling in different contexts. This also holds true for liver cancer.

## 2. Overview of TGF-β Signaling

TGF-β is a prototype of the TGF-β family cytokines, which is composed of 33 members in mammals according to their genomes [25]. Based on sequence and structural similarity, these proteins are roughly divided into two subfamilies [26,31]. TGF-β, Activin, Nodal and Lefty constituent the TGF-β subfamily, while the majority of bone morphogenetic proteins (BMPs) and growth and differentiation factors (GDFs) fall into the BMP subgroup. Among them, TGF-β was the first to be purified and cloned in the 1980s, and is also the most extensively studied [32].

The TGF-β family ligands are secreted as dimers by disulfide linkage [33,34]. Some of them are trapped by extracellular binding proteins and kept in an inactive state. For example, Dan/Cerberus, Tsg, noggin and chordin are capable of binding to and inactivating BMPs, whereas FST and FSTL1/3 could target not only BMPs, but also Activin and Nodal, in similar manners [35]. Unlike them, the mature TGF-β dimers are associated with their pro-domains and stored in the extracellular matrix (ECM), at the latent state [34,35]. Therefore, TGF-β needs release and activation before binding to its cognate transmembrane receptors [34].

**Figure 1:**
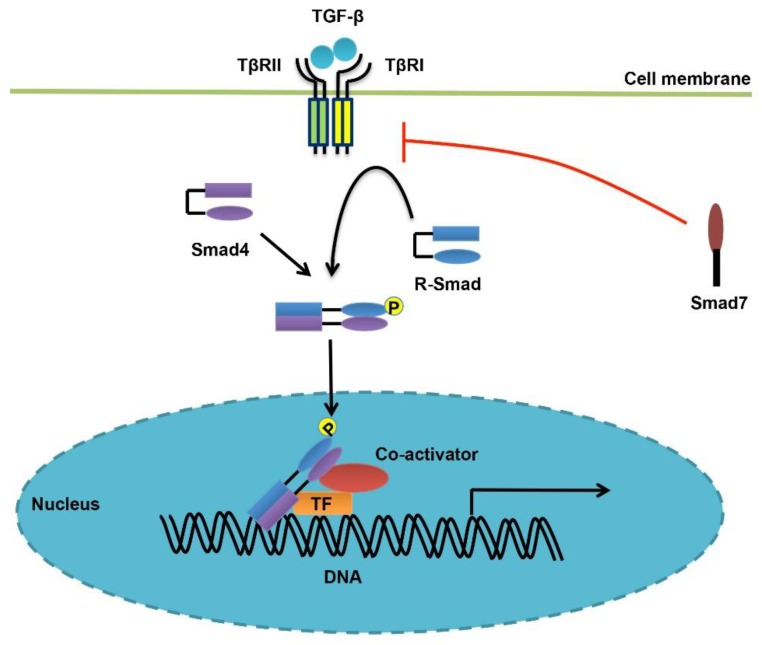
Overview of TGF-β/Smad signaling. Once associated with TGF-β ligands, the constitutively active TβRII forms a complex with and phosphorylates TβRI, leading to activation of its kinase activity. Then, TβRI proceeds to phosphorylate R-Smads (Smad2/3) at the extreme C-terminal SXS motif, causing their oligomerization with Smad4, translocation into the nucleus, binding to DNA, and finally regulation of target gene transcription, in concert with other transcription factors and co-regulators. In addition, the inhibitory Smad Smad7 exerts a critical regulatory effect over TGF-β/Smad signaling, in a feedback manner and via multiple mechanisms.

There are a total of five type II receptors (TβRII, ActRII, ActRIIB, BMPRII, and AMHRII) and seven type I receptors (ALK1-7) for TGF-β family cytokines, and both types of receptors bear intrinsic serine/threonine kinase activity [36,37]. As for TGF-β, the ligand dimer first binds to a pair of type II receptors (TβRII) believed to be constitutively active. Then, two type I receptors (TβRI/ALK5) are recruited to form a stable receptor complex, in which TβRI is phosphorylated at the glycine/serine-rich GS domain by TβRII. TβRI then propagates and amplifies theTGF-β signal by recruiting receptor-regulated Smad proteins (R-Smads, Smad2 and Smad3 for TGF-β) and phosphorylating them at the extreme C-terminal SXS motif. Activated R-Smads continue to form an oligomeric Smad complex with the common Smad (Co-Smad, Smad4), and they together translocate into the nucleus, bind to DNA and regulate the expression of target genes (Figure 1). Most BMPs utilize Smad1/5/8 as their R-Smads, but transmit signals similar to TGF-β, from a biochemical perspective [38,39,40].

Structurally, both R-Smads and Smad4 contain two conserved mad homology (MH) domains [37,41,42]. The N-terminal MH1 domain is mainly responsible for DNA binding (except for the full-length of Smad2), whereas the C-terminal MH2 domain mediates various protein-protein interactions including the receptor-Smad interaction, the oligomeric Smad complex formation, and interactions of Smads with other transcription factors, transcriptional co-factors or regulators. The two MH domains are connected with a linker region that differs in length and amino acid sequence. However, the linker region contains some conserved motifs that are targeted by various regulators and post-translational modifications (PTMs) especially phosphorylation, delicately adjusting the signaling activity of Smads [41]. Smad6 and Smad7 are the only two members that constitute a third Smad subfamily, namely inhibitory Smads (I-Smads) [43,44]. Unlike R-Smads and Smad4, I-Smads only contain a conserved MH2 domain that mediates their associations with the receptors or R-Smads. In this way, they inhibit TGF-β/BMP signaling [30,43,45]. Among them, Smad6 specifically antagonizes BMP signaling, whereas Smad7 acts as a general inhibitor for both TGF-β and BMP pathways [43].

**Figure 2:**
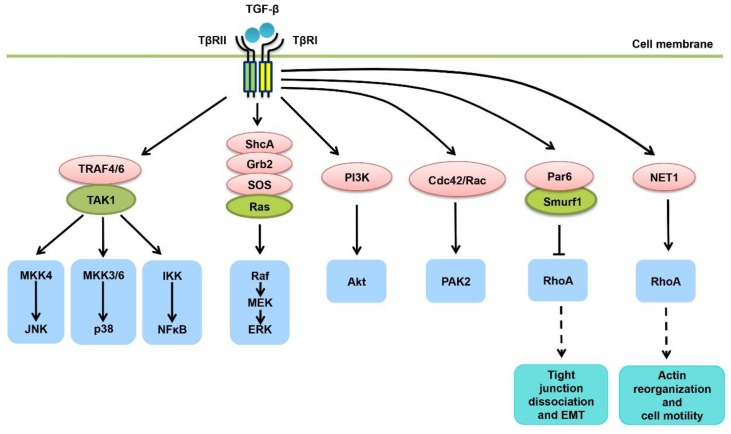
TGF-β activates versatile non-Smad pathways. Ligand-bound TGF-β receptors activate TAK1 by associating with TRAF4/6, resulting in activation of the MKK4-JNK, MKK3/6-p38 or IKK-NFκB signaling cascades. TβRI is able to phosphorylate the adaptor protein ShcA at both serine and tyrosine residues, causing activation of Ras/ERK MAPK signaling. TGF-β receptors could also interact with p85, the regulatory subunit of PI3K, leading to activation of PI3K/Akt signaling. In addition, TGF-β receptors may induce a rapid activation of the small GTPases Cdc42/Rac, and subsequent activation of PAK2 kinase. RhoA has been shown as an important mediator of TGF-β in different contexts, contributing to actin reorganization and cell motility. Intriguingly, TGF-β receptors could induce a localized and ubiquitin-mediated degradation of RhoA in tight junctions, through TβRII-induced phosphorylation of Par6 and recruitment of the E3 ubiquitin ligase Smurf1, leading to EMT induction.

As aforementioned, non-Smad signaling molecules play a crucial role in understanding TGF-β-elicited signal transduction network and the TGF-β actions in a given context [29]. These non-Smad pathways could emanate from either the type II or the type I receptors, depending on the proteins associated with them (Figure 2). Intriguingly, although generally regarded as serine/threonine kinases, TGF-β receptors are actually dual-specificity kinases, exhibiting tyrosine-phosphorylating activity in some circumstances [36,37]. Upon TGF-β stimulation, the adaptor protein ShcA associates with TβRII that is phosphorylated by Src at tyrosine residues, or with TβRI for a higher affinity as described in another study [46,47]. TβRI induces both tyrosine and serine phosphorylation in ShcA, which then forms a complex with Grb2 and Sos, channeling the TGF-β signal in ERK MAPK activation. In addition, two RING domain-containing E3 ubiquitin ligases, TRAF6 and TRAF4, associate with activated TβRI and undergo intracellular polyubiquitination at lysine 63, facilitating recruitment of TAK1 and subsequent activation of JNK/p38 MAPKs and NFκB signaling [48,49,50]. PI3K/Akt signaling is another critical pathway that could be quickly induced by TGF-β treatment in different cell types, contributing to TGF-β-mediated cell survival, EMT and other tumor-promoting effects [51,52]. p85, the regulatory subunit of PI3K, has been found constitutively associated with TβRII, and also with TβRI in response to TGF-β stimulation [53]. Interestingly, TRAF6 polyubiquitination has been suggested to be involved in TGF-β-mediated PI3K/Akt activation [54]. Furthermore, small GTPases including RhoA, Cdc42 and Rac are also downstream of TGF-β receptors in some contexts, regulating cytoskeleton reorganization, cell motility or gene transcription [52,55]. Among them, Cdc42 and Rac are capable of activating PAK2 kinase and regulating cytoskeletal organization, by associating with TGF-β receptors [56]. Although being repeatedly shown to mediate TGF-β cellular actions, RhoA has been alternatively found to be degraded by TGF-β receptors in tight junctions, wherein TβRII phosphorylates Par6 to recruit the E3 ubiquitin ligase Smurf1, promoting tight junction dissociation and EMT [52,57]. Moreover, TGF-β may also activate Stat3 signaling in some cell types including hepatic stellate cells, via intracellular tyrosine kinase JAKs [58].

To add another layer of complexity, TGF-β-activated non-Smad proteins are likely to regulate the signaling activity of Smads, forming various feed-forward loops [30]. In this regard, some kinases are able to phosphorylate Smads directly. For instance, MAPKs have been found to induce linker region phosphorylation of Smads in various contexts [41,52]. We reported that PAK2 acts as a novel inhibitor of TGF-β signaling in epithelial cells, by phosphorylating Smad2 (and probably also Smad3) directly [59].

## 3. Alterations of TGF-β Signaling in Liver Cancer Revealed by High-Throughput Studies

Rewiring of the intracellular signaling network features tumorigenesis and malignant progression. In recent years, advances in various “omics” technologies and relevant investigations have furthered our understanding of how TGF-β signaling alterations contribute to initiation and progression of live cancer, from a high-throughput perspective [6,60,61].

At the genomic level, a recent study using a “pan-cancer cohort” that involves 9125 tumor samples across 33 cancer types in TCGA (The Cancer Genome Atlas) has revealed that 39% of the samples bear genomic alterations of the TGF-β family pathways [62]. Of those, mutational hotspots have been identified in genes encoding TβRII, Smad4, and Smad2, especially in the gastrointestinal cancers including esophageal carcinoma, colon adenocarcinoma and pancreatic cancer. These results are in line with the notion that TGF-β mediates a critical tumor-suppressive pathway [8,10,12]. Although the genomic aberration rate is relatively lower in liver cancer, a study of 161 candidate diver genes in HCC showed that the TGF-β pathway is altered at a rate of about 5% [63]. It was recently found that 38% of the investigated HCC samples exhibit at least 1 gene mutation in the TGF-β pathway [64].

In fact, TGF-β plays a dichotomous role in liver cancer [9,10]. An early comparative functional genomic study suggested that there could be two distinct groups of TGF-β-responsive genes [65]. The early TGF-β signature genes are closely related to the tumor-suppressive functions of TGF-β, including cell cycle arrest and apoptosis, whereas the late signature is relevant to cancer progression, being associated with overall poor clinical outcomes in HCC patients. Intriguingly, analyses of a TCGA HCC cohort using comprehensive integrated “omics” approaches have identified three different TGF-β signatures [64]. Activated TGF-β signaling in one group of the HCC samples is associated with liver fibrosis, inflammation, and cancer development. Interestingly, another group of HCC with inactivated TGF-β signaling is able to escape from TGF-β-mediated tumor inhibition, and exhibit an even worse survival rate than patients with activated TGF-β signature [64]. In another study, TGF-β-mediated Wnt pathway activation has been found in a subclass of HCC samples, which are poorly differentiated and could form larger tumors [66]. Quantitative proteomic study of a cohort of early HCC samples demonstrated that upregulation of some of the genes in the TGF-β pathway is associated with microscopic vascular invasion and poor prognosis [67].

Together, the above results have demonstrated obvious alterations of TGF-β signaling in liver cancer development, at levels from DNA and RNA to protein. Although gene expression variation of TGF-β signaling components has been connected with liver cancer progression, as most notably exemplified by TGF-β upregulation and TβRII downregulation [9,68], it is now apparent that signaling robustness and duration play a pivotal role in determining the versatile and context-specific functions of TGF-β [30,36,37,43]. With regard to this, TGF-β signaling is finely tuned in various contexts by plenty of regulators and mechanisms. It is rational that the contextual expression level and activity of different regulators could profoundly affect the actions of TGF-β [24,25,26]. In this review, we summarize the current understanding of the contextual regulatory mechanisms of TGF-β signaling in liver cancer, and how they channel, skew and even switch the functions of TGF-β.

## 4. Enhanced Bioavailability of TGF-β Ligand in Liver Cancer

**Figure 3:**
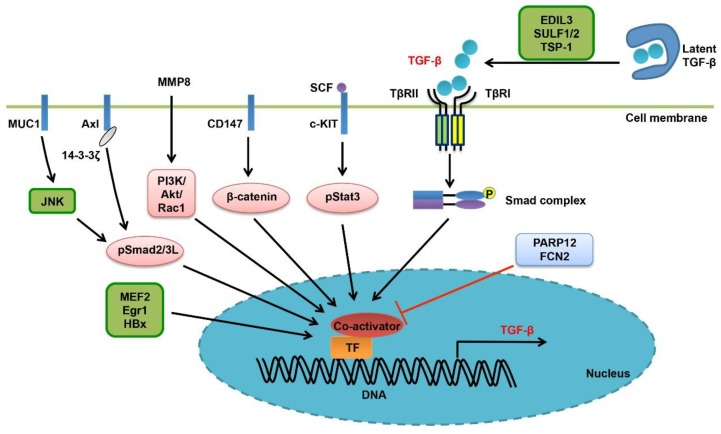
Bioavailability of the TGF-β ligand in liver cancer. In the resting state, the mature TGF-β ligand dimer is trapped and stored in the ECM. TGF-β is not able to associate with its cognate cell membrane receptors until activated by integrins, proteases or other proteins. EDIL3, SULF1/2 and TSP-1 are involved in this process. Some extracellular factors and membrane receptors, such as SCF/c-KIT, Axl, CD147, MUC1 and MMP8, are able to induce TGF-β gene expression through different intracellular pathways. TGF-β expression is also under the control of TGF-β/Smad signaling, forming an autoregulatory loop. In addition, some other proteins like MEF2, Egr1, HBx, PAPR12 and FCN2 may regulate TGF-β transcription either positively or negatively.

Ligand bioavailability is one of the vital factors regulating the signaling activity and function of TGF-β family cytokines [34,35]. It is determined by the expression level, release into and storage in the ECM, activation process, and receptor binding of the ligands (Figure 3). Overall, these processes are finely controlled in both normal hepatocytes and cancerous cells.

TGF-β is synthesized as a ~50 KDa protein that includes both the C-terminal mature TGF-β fragment and an N-terminal pro-domain [34,69]. The full TGF-β peptide undergoes dimerization and folding within the endoplasmic reticulum, and subsequently cleavage by furin-type enzymes in the Golgi apparatus. Interestingly, the pro-domain exhibits high affinity towards the mature TGF-β peptide. They associate with one another even after release into the ECM, forming the small latency complex (SLC) and preventing the mature TGF-β dimer from binding to their receptors. In fact, in most cases, this SLC would be linked to LTBPs (LTBP1/3/4) by a pair of disulfide bonds, creating the large latency complex (LLC) and depositing TGF-β in ECM. The latency and storage of TGF-β not only maintain basal TGF-β signaling under physiological conditions, but also provide a mechanism to soon motivate a large amount of ligands when necessary, such as in embryonic development and in some pathologic circumstances like advanced cancers [34,35,69].

Physical and chemical cues have been implicated in activation of latent TGF-β, such as ROS oxidation, acid or basic pH, heat denaturation, and physical shear or stirring [34]. However, biological factors seem to play a dominant role in vivo. First of all, transmembrane integrin-mediated TGF-β activation has been well established [34,69,70]. In this regard, αvβ6 and αvβ8 are the most extensively investigated. They are able to interact with the RGD motif (arginine-glycine-aspartic acid) in the TGF-β pro-domain, or with that in LTBPs, leading to conformational change of the latency complex and subsequent exposure or release of the mature TGF-β. Matrix metalloproteinases (MMPs) have been shown in some contexts to facilitate the effects of integrins, such as MMP-2/9 for αvβ6 integrin and MMP-14 (or MT1-MMP) for αvβ8 integrin. Besides, several other integrins including αvβ1, αvβ3, αvβ5, α5β1, α8β1, and αIIbβ3 can also recognize the RGD motif, thereby being implicated in TGF-β activation in different circumstances. Second, various proteases have been suggested to activate TGF-β, including cysteine proteases like calpain, aspartyl proteases like cathepsin D, serine proteinases like kallikreins and plasmin, and MMPs [34,69]. Third, some cell surface proteins without enzymatic activity may also promote TGF-β activation in other ways, such as Thrombospondin 1 (TSP1) and F-spondin (spondin-1) [35]. It is worth noting that many of the above proteins regulating ligand bioavailability are transcriptionally induced by TGF-β, generating various feedback or feed-forward regulatory loops [30,69].

Although kept at a basal level in normal liver, the expression of TGF-β is quickly induced upon liver injury, and maintained high in hepatitis, liver fibrosis, cirrhosis and liver cancer [9,10,12]. It has been found that TGF-β is highly expressed in HCC tissue samples when compared with those in normal tissues [71]. High-throughput study of early HCC tissue samples via proteomics revealed that the TGF-β protein is highly expressed in invasive HCC tissues [67]. The serum TGF-β level also increases dramatically in HCC patients [19,20,72,73]. Importantly, the enhanced TGF-β level is associated with advanced stage and poor prognosis and outcomes, making it a promising diagnostic and prognostic maker.

At the transcriptional level, enhanced autoregulation of TGF-β expression is one of the key features of advanced HCC (Figure 3). Overexpression of a dominant-negative Smad2 mutant, Smad2 (3A), in Huh-7 HCC cell line, leads to activation of Smad3/Smad4 signaling and transcriptional induction of TGF-β, PAI-1 and VEGF [74]. Axl, a tyrosine kinase receptor, has been reported to associate with 14-3-3ζ, induce JNK-mediated phosphorylation of the linker region of TGF-β-activated Smad2/3, generate linker- and C-terminus-double phosphorylated R-Smads, and then promote the expression of target genes relevant to invasion and metastasis including the TGF-β gene itself [75]. TGF-β induces MMP8 gene expression through the PI3K/Akt/Rac1 signaling in HCC cells, and reciprocally, MMP8 could also activate the PI3K/Akt/Rac1 pathway to promote TGF-β expression. This positive feedback loop makes sense as to cancer cell EMT and malignant progression [76]. Similarly, TGF-β induces myocyte enhancer factors 2 (MEF2) expression in HCC cells in a PI3K/Akt-dependent manner, and MEF2 in turn acts as a transcription factor to activate TGF-β transcription, creating another positive feedback loop and promoting HCC cell motility [77]. Interestingly, TGF-β also forms tumor-promoting positive feedback loops with the stem cell factor (SCF) or the membrane protein CD147. SCF is transcriptionally upregulated by TGF-β/Smad2 signaling in HCC cells, and induces TGF-β expression by activating the JAK1/Stat3 signaling [78]. TGF-β-induced CD147 could also promote TGF-β signaling in liver cancer by enhancing TGF-β gene transcription and by facilitating the maturation of latent TGF-β [79].

Some other factors also contribute to the high expression level of TGF-β in liver cancer (Figure 3). Early growth response factor 1 (Egr1) is an oncogene that is upregulated in liver cancer. It enhances TGF-β expression level and activates Smad signaling in HCC [80]. PARP12, a mono-ADP-ribosyltransferase, has been shown to inhibit HCC cell invasion and metastasis. Deficiency of PARP12 could increase TGF-β expression, independently of its enzymatic activity. Furthermore, hepatitis virus B (HBV) and hepatitis virus C (HCV) have been shown to promote liver tumorigenesis by rewiring TGF-β signaling via multiple mechanisms (Table 1). With regard to it, the HBV X protein (HBx) is able to switch TGF-β from a tumor suppressor to a promoter [81]. As one the mechanisms, HBx increases TGF-β expression in HCC cells [82]. Mucin1 (MUC1), a transmembrane glycoprotein and an oncogene that promotes HCC cell motility, is able to induce autocrine TGF-β secretion by promoting JNK-mediated linker region phosphorylation of Smad2/3 [83,84].

In addition to the transcriptional control, the activation, presentation and stability of TGF-β ligand are also subject to regulation in liver cancer (Figure 3) [34]. The expressions of several integrin subunits, including the α1, α2, α3, α5, α6, and β1 chains, are upregulated in HCC cells and associated with cancer cell invasion and poor prognosis, in line with the notion that they act as activators of TGF-β ligand [85]. Of relevance, EDIL3 promotes EMT and liver cancer malignant progression by interacting with integrins and facilitating TGF-β activation [86]. Sulfatase 1 (SULF1) modulates the activity of heparan sulfate proteoglycans (HSPGs), the type III TGF-β receptor, by removing sulfate residues. In this way, transgenic expression of SULF1 in mice resulted in release of TGF-β from cell surface and enhancement of TGF-β signaling, thereafter promoting HCC cell EMT and invasion [87]. In HCV core protein transgenic mice, the active TGF-β level also increases and TGF-β/Smad signaling is reinforced in both hepatocytes and in stroma, by engaging thrombospondin-1 [88]. Unlike Stat3 that is usually oncogenic, Stat5 is a tumor inhibitor. Intriguingly, Stat5 has been shown to interact with TGF-β, and Stat5 deficiency in mice leads to stabilization of mature TGF-β, promoting carbon tetrachloride (CCl_4_)-induced liver fibrosis and HCC development [89]. Finally, Ficolin-2 (FCN2) was found to inhibit liver cancer by decreasing the expression level of TGF-β, yet the mechanism remains unknown [90].

## 5. Deregulation of TGF-β Receptors

TGF-β receptors are critical hotspots for intense regulation [36,37,100]. Their deregulation has been associated with evasion of TGF-β-mediated growth inhibition of liver cancer and their malignant progression [10,16,51].

Although TGF-β receptors are rarely mutated at the genomic level, decreased TβRII expression level has been shown to desensitize HCC cells to TGF-β-induced growth inhibition. An early study showed that reduced TβRII expression, and also the expression of type I and type III TGF-β receptors, are connected with N-nitrosodiethylamine (DEN)-initiated and phenobarbital (PB)-promoted liver tumorigenesis in rat [101]. Interestingly, TβRII expression is downregulated in metastatic HCC tissues and cell lines, and is associated with larger tumor size, poor differentiation, portal vein invasion, intrahepatic metastasis (IM), and shorter recurrence-free survival [102,103]. These results are in accordance with those obtained from TβRII gene-targeting studies. Inactivation of TGF-β signaling by TβRII knockout is capable of enhancing hepatocyte proliferation and promoting liver regeneration [104,105]. Heterozygous deletion of TβRII and transgenic expression of a dominant-negative TβRII in mouse liver render hepatocytes less sensitive to TGF-β-induced growth-inhibitory effect and potentiate DEN-induced liver tumorigenesis [14,106]. Soluble TβRII, the extracellular domain of TβRII, has been suggested to inhibit rat liver tumorigenesis by trapping TGF-β ligand [107]. In addition, when TGF-α is simultaneously overexpressed in the liver, loss of TGF-β signaling by TβRII deletion in mouse hepatocytes exhibits a more obvious proliferation-promoting effect in DEN-induced HCC development, suggesting a synergistic effect [103]. Together, the above results indicate that TβRII plays a pivotal role in TGF-β-induced liver cancer inhibition. Meanwhile, other evidence has demonstrated that TβRII is also involved in TGF-β-mediated oncogenic effects in liver cancer, such as EMT, invasion and metastasis. With respect to this, PDGF-B has been shown to promote the development of liver fibrosis and tumorigenesis by upregulating TβRII gene expression [108]. IQ motif-containing GTPase activating protein 3 (IQGAP3) promotes TGF-β signaling to enhance HCC cell EMT, migration and invasion. In accordance with this, IQGAP3 is highly expressed in clinic HCC tissues and is associated with aggressive cancer features [109]. In contrast, IQGAP1 associates with TβRII upon TGF-β stimulation in hepatic stellate cells (HSCs), and promotes Smurf1-mediated TβRII ubiquitination and degradation, thereby inhibiting differentiation of HSCs into myofibroblasts and preventing metastatic growth of lung cancer cells and colon cancer cells [110]. Why IQGAP1 and IQGAP3 play distinct roles in liver cancer remains an open albeit interesting question.

**Figure 4:**
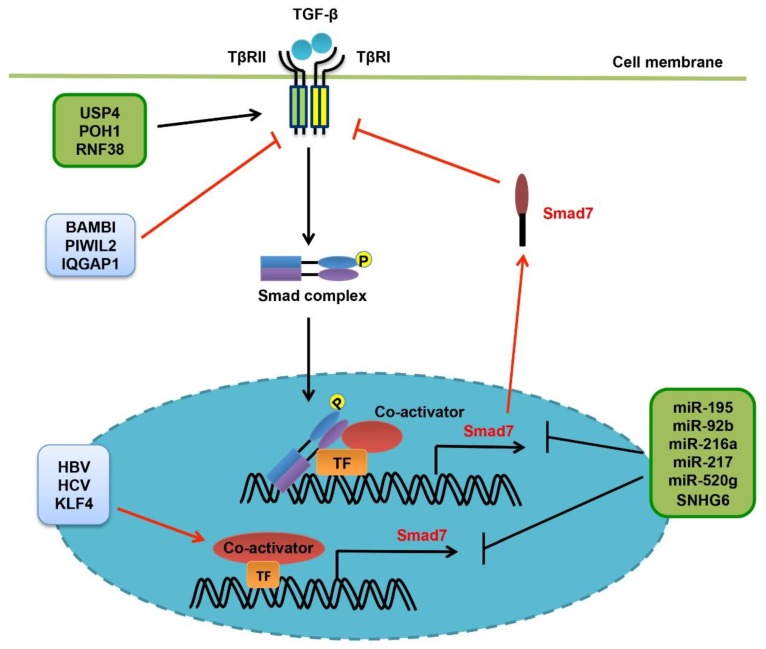
Regulation of TGF-β receptors. Smad7 has been recognized as a central hub in regulating TGF-β receptors, acting via feedback loops and multiple mechanisms. Therefore, regulation of Smad7 gene expression by HBV/HCV, the transcription factor KLF4 or some miRNAs, is rational to modulate the activity of TGF-β receptors in liver cancer. Importantly, deregulated Smad7 leads to altered TGF-β signaling, which is closely associated with liver tumorigenesis, in a context-dependent manner. In addition, the deubiquitylating enzymes USP4 and POH1 could directly interact with and stabilize TβRI. RNF38 also enhances TβRI protein stability by targeting AHNAK. On the contrary, BAMBI, PIWIL2 and IQGAP1 are able to inhibit the activity of TGF-β receptors.

The signaling activity and protein stability of TβRI are finely controlled and well documented (Figure 4). Smad7 has been established to play a central role in regulating TβRI activity and TGF-β signaling, via different mechanisms in varying contexts [43,44,111]. Smad7 was firstly identified to form a stable complex with TGF-β receptors upon ligand stimulation, compelling the recruitment and activation of R-Smads [112,113]. Second, Smad7 is able to recruit some WW-HECT type E3 ubiquitin ligases including Smurf1/2, NEDD4-2 (NEDD4L) and WWP1/Tiul1, leading to TβRI poly-ubiquitination and degradation [43,44,114,115]. Third, Smad7 may also recruit the phosphatase PP1 by interacting with its regulatory subunit GADD34, promoting TβRI inactivation [116]. Fourth, Smad7 could also function at the Smad protein level and the transcription level. We have reported that TGF-β stimulation induces association of Smad2/3 with either Smad4 to relay TGF-β signal or Smad7 to terminate TGF-β signaling [45]. Smad7 achieves this by competing with Smad4 to interact with R-Smads, or by promoting NEDD4L-mediated ubiquitination and degradation of activated Smad2/3 [45]. Smad7 was also suggested to target Smad4 for degradation by recruiting some WW-HECT type E3 ligases, relying on receptor-activated R-Smads [117]. Furthermore, Chen and colleagues found that Smad7 retains in the nucleus in some cancer cell lines even after TGF-β treatment, and inhibits TGF-β-induced transcriptional responsiveness by interfering with the R-Smad-Smad4-DNA complex formation [118]. The nuclear protein YY1 is able to potentiate the transcription-repressive activity of Smad7 by recruiting the histone deacetylase HDAC1 [119]. Collectively, the multiple and redundant mechanisms would guarantee efficient inhibition of TGF-β signaling by Smad7. Not surprisingly, Smad7 gene transcription is controlled by various extracellular cues, intracellular signaling pathways and nuclear transcription factors. Smad7 protein stability is well balanced by ubiquitin ligases and deubiquitinating enzymes [43,44]. Smad7 activity is also regulated by other post-translational modifications (PTMs) including phosphorylation, acetylation and methylation, and also by some protein regulators without or independently of enzymatic activity. In this regard, TSC-22, SIK, Cas-L, STRAP, BAMBI and others, fine-tune Smad7 activity and TGF-β signaling [43,44,120,121,122,123,124].

Smad7 exhibits a biphasic role in liver cancer development, like that of TGF-β. Overexpression of Smad7 has been shown to block TGF-β-mediated cytostatic effects in several HCC cell lines [125,126]. Hepatocyte-specific expression Smad7 in transgenic mice promoted compensatory proliferation in hepatocytes [126]. Ectopic expression of Smad7 in mice dramatically enhanced liver tumorigenesis induced by HRAS (G12V), in the presence or absence of c-Myc overexpression [127]. Nuclear Smad7 is able to attenuate TGF-β-mediated apoptosis of HCC cells [118]. Interestingly, HBV could enhance the Smad7 expression level by downregulating miR-15a that directly targets Smad7 in HepG2 HCC cells, attenuating TGF-β-induced apoptosis [94]. Similarly, HCV core protein also promotes HepG2 cell proliferation and chemoresistance by enhancing Smad7 expression [95]. Reppression of Smad7 by miR-195 reinforces TGF-β-mediated growth inhibition in liver cancer [128]. In accordance with these observations, the expression of Smad7 has been found upregulated in some HCC tissue samples when compared with that in normal tissues [126,129].

On the other hand, mounting evidence has pointed out that Smad7 may act as a tumor suppressor in liver cancer by interfering with the oncogenic effects of TGF-β or other tumor-promoting pathways. Smad7 gene knockout or its hepatocyte-specific deletion in mice has been reported to enhance proliferation but decrease apoptosis in hepatocytes, in which TGF-β signaling is elevated. This renders hepatocytes more sensitive to DEN-induced tumorigenesis [130,131]. Interesting, the tumor-promoting effects of Smad7 are linked to activation of NFκB and Stat3 signaling in these mouse models [130,131]. Consistently, Smad7 overexpression in mouse liver resulted in tumor suppression in HRAS (G12V)-transgenic mice with loss of p53 or activation of YAP/TAZ [127], suggesting that the seemingly contradictory roles of Smad7 in liver tumorigenesis could be determined by the genetic contexts. In advanced liver cancer, Smad7 is able to inhibit TGF-β-induced expression of Snail, a master transcription factor in EMT, alleviating HCC formation in mouse liver [127]. Smad7 has been found to interfere with TGF-β-mediated expression of TM4SF5 expression, EGFR pathway activation and subsequent EMT in both normal hepatocytes and HCC cell lines [132]. Smad7 could also abolish TGF-β-elicited DNMT3β expression in Huh7 HCC cells, leading to promoter demethylation and gene activation of CD133, which is able to drive liver tumorigenesis. By this mechanism, Smad7 attenuates tumor growth in vitro and tumor formation in vivo [133].

In keeping with its critical roles in regulating TGF-β signaling and in liver cancer, Smad7 gene expression is tightly regulated in liver cancer by contextual transcription factors and co-factors (Figure 4). The long non-coding RNA X-inactive specific transcript (XIST) and the microRNA 92b (miR-92b) could directly interact with and repress each other. XIST inhibits HCC cell proliferation and metastasis, whereas miR-92b promotes EMT and invasion of HCC cells by targeting Smad7 [134]. miR-216a/217 promote EMT, drug resistance and recurrence of liver cancer by targeting Smad7 and PTEN, which inhibit TGF-β and PI3K/Akt signaling, respectively [135]. miR-520g also inhibits Smad7 gene expression to enhance TGF-β-mediated HCC cell EMT and motility [136]. Interesting, snoRNA host gene 6 (SNHG6), which encodes a snoRNA, has been shown to promote HCC progression by decreasing Smad7 expression and enhancing TGF-β signaling [137]. In addition, protein regulators are also involved in regulating Smad7 gene expression. Neural precursor cell expressed, developmentally downregulated 9 (NEDD9) is highly expressed in human HCC tissues, and promotes HCC cell EMT, stemness and metastasis by alleviating Smad7-mediated inhibition of TGF-β signaling [138]. On the contrary, KLF4 protein could inhibit TGF-β-induced EMT and HCC progression by inducing Smad7 gene expression, directly or via inhibiting KLF11 [139,140]. However, the tumor-suppressive KLF4 is downregulated during HCC development [139]. Two independent studies have demonstrated that the expression level of Smad7 is significantly reduced in some cohorts of liver cancer tissues when compared with normal tissues [130,131]. Although contradicting with those obtained in two other studies [126,129], these results suggest that the expression and function of Smad7 in liver cancer are highly reliant on contexts, such as developing stage and cancer subclassification.

Besides Smad7, other proteins are also involved in liver cancer by regulating TGF-β receptors (Figure 4). The ubiquitin-specific protease 4 (USP4) has been identified as a deubiquitinase for TβRI, and coordinates Akt- and TβRI-mediated signal transduction to promote cell motility in breast cancer [141]. Similarly, USP4 is able to stabilize TβRI and promote cell invasion and metastasis in liver cancer [142]. POH1 belongs to the JAMM domain metalloprotease family of DUBs. It enhances the protein stability of TβRI and caveolin-1, thereby promoting HCC metastasis [143]. PIWIL2 has been shown to promoting TβRI degradation and promote proliferation of HepG2 HCC cells, possibly by interfering with the HSP90-TβRI complex formation [144]. Intriguingly, the RING finger protein 38 (RNF38), an E3 ubiquitin ligase, has been recently shown to be overexpressed in HCC tissues and associated with malignant progression. It may achieve this by enhancing the TβRI expression level and Smad signaling activity [145]. BAMBI, a pseudoreceptor of TGF-β receptors, inhibits TGF-β signaling by interfering with the functional receptor complex formation or the receptor-Smad interaction [120]. In liver cancer, BAMBI could promote tumorigenesis by alleviating TGF-β-mediated growth inhibition [146].

## 6. Regulation of Smad Proteins

Smad proteins, including R-Smads and Smad4, mediate both the tumor-inhibitory and promoting effects of TGF-β [10,26]. Understanding how cancer cells evade Smads-mediated cytostatic effects and, on the other hand, take advantage of their oncogenic potential, is one of the crucial keys to unlock the mysteries of TGF-β in liver cancer. Embryonic liver fodrin (ELF), a β-spectrin, acts as an adaptor protein for Smad3 and Smad4 by facilitating their nuclear translocation and transcriptional activity [147]. ELF deficiency in mice led to disruption of TGF-β signaling, and up to 40% of the ELF^+/-^ mice spontaneously developed HCC [148]. ELF associates with either CDK4 or Smad3, in both competitive and TGF-β-dependent manners, thereby compelling the growth-inhibitory effect of TGF-β [149]. Of relevance, PRAJA, an RING finger protein with E3 ubiquitin ligase activity, has been reported to associate with ELF in liver cancer cells, promoting ELF ubiquitination and degradation [150]. In line with these observations, ELF is downergulated while PRAJA is upregulated in liver cancer tissues [150].

**Figure 5:**
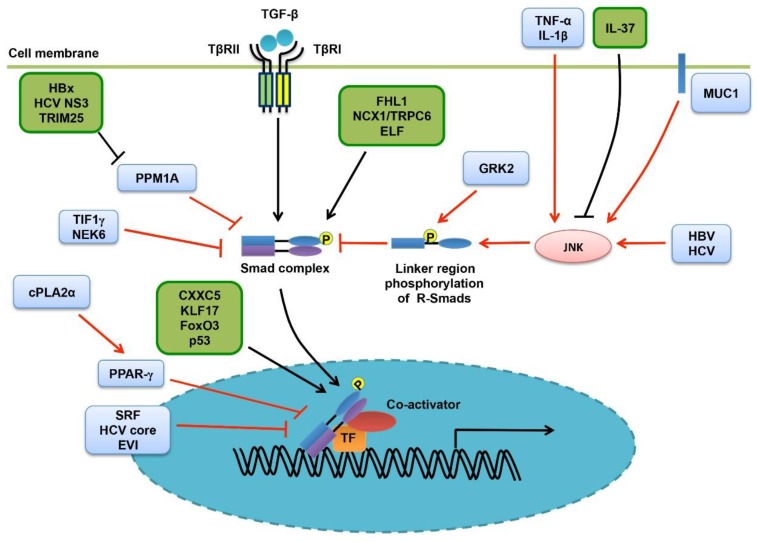
Controlling the activity of Smad proteins in liver cancer. The signaling activity and transcriptional activity of Smads are well controlled by PTMs. FHL1 promotes Smad activity by inducing their C-terminal phosphorylation, independent of TGF-β receptors. In contrast, the phosphatase PPM1A specifically targets Smad2/3 by counteracting TGF-β-induced phosphorylation. Interestingly, HBx, HCV NS3 and TRIM25 could enhance Smad signaling by promoting PPM1A degradation. Extracellular TNF-α and IL-1β, the membrane-bound protein MUC1, and also HBV/HCV, are able to promote JNK-mediated linker region phosphorylation in R-Smads, thereby impelling Smad signaling. Differently, IL-37 may promote the signaling activity of R-Smads by reducing their linker region phosphorylation. Furthermore, GRK2 could also phosphorylate R-Smads at their linker regions in liver cancer. Unlike the above, both TIF1γ and NEK attenuate TGF-β signaling by inhibiting Smad4. NCX/TRPC6 and ELF are required for Smad signaling by regulating cellular calcium flux or acting as an adaptor protein. In the nucleus, CXXC5, KLF17, FoxO3 and p53 are necessary for TGF-β-activated Smads to exert their transcription-regulating activity towards certain genes, depending on contexts and cancer stages. However, cPLA2α-activated PPAR-γ, SRF, EVI and the HCV core protein are able to inhibit the transcriptional activity of Smad proteins.

Smad proteins are subjected to delicate regulation by various PTMs in liver cancer (Table 2 and Figure 5). G-coupled receptor kinase 2 (GRK2) has been demonstrated transcriptionally induced by TGF-β/Smad signaling, and its protein product interacts with and phosphorylates R-Smads at their linker regions, thereby impairing Smads-mediated cytostatic effects [151]. The four-and-a-half LIM proteins (FHL1/2/3) are frequently downregulated in HCC tissues and exert a tumor-suppressive role. Intriguingly, FHL1 could trigger CK1δ-mediated and TGF-β receptor-independent phosphorylation of R-Smads, activating their transcriptional activity and anti-proliferative ability [152]. However, it is worth noting that PARP12, a mono-ADP-ribosyltransferase, was recently found to stabilize FHL2 and decrease the TGF-β1 expression level, inhibiting HCC cell EMT and metastasis [81]. Moreover, NIMA-related kinases 6 (NEK6) may exert a tumor-promoting effect in liver cancer by interacting with Smad4 and antagonizing the tumor-inhibitory effect of TGF-β [153]. Taken into account the fact that NEK6 transcription is downregulated by TGF-β in liver cancer cells, the double-negative feedback loop between TGF-β signaling and NEK6 is of significance in driving liver tumorigenesis [153].

On the other hand, the tumor-promoting activity of TGF-β-activated Smads is also under delicate regulation (Table 2 and Figure 5). With regard to this, linker region phosphorylation of Smads has been suggested to play an important role in adjusting the signaling activity of R-Smads, rendering them to promote chronic liver diseases including hepatitis, fibrosis, cirrhosis, and also liver tumorigenesis [154]. Chronic liver inflammation could be a prerequisite for the development of most liver cancers [12,16]. In fact, the pro-inflammatory cytokines TNF-α and interleukins-1β have been found to induce JNK-mediated linker phosphorylation of Smad3 (pSmad3L), leading to enhanced c-Myc expression and hepatocyte proliferation and reduced levels of pSmad3C signaling and p21 expression [92,154]. HCV could promote HCC development by shifting the TβRI/pSmad3C/p21 tumor-suppressive pathway to JNK/pSmad3L/c-Myc oncogenic signaling [92]. In addition, HCV also induced JNK-mediated linker phosphorylation of pSmad2C to generate pSmad2L/C, contributing to HCC development [96]. Similarly, HBx promotes HCC by altering the phosphorylation pattern of Smad3 [93]. Shifting of the Smad3 phosphorylation pattern has been verified in HBV- and HCV-related HCC tissues samples [92,93]. Consistently, inhibiting JNK kinase activity in this liver cancer model switched Smad3 signaling from oncogenic to tumor-suppressive [155]. Interestingly, Mucin1 has been suggested to associate with JNK directly, convert TGF-β-induced pSmad3C/p21 signaling to the pSmad3L/c-Myc signaling, and promote HCC progression [83,156]. On the contrary, IL-37 was found to inhibit Smad3-mediated oncogenic effects by inducing a conversion from pSmad3L/c-Myc signaling to pSmad3C/p21 signaling [157]. However, this effect is alleviated in human HCC tissues and cell lines due to reduced IL-37 expression [157]. In addition to the linker region, phosphorylation of the C-terminus of R-Smads is also regulated. PPM1A is a critical phosphatase targeting TGF-β-induced pSmad2/3 phosphorylation, terminating Smad signaling [158]. Intriguingly, both HBx and the HCV nonstructural protein 3 (NS3) are capable of promoting ubiquitination and degradation of PPM1A, thereby promoting Smad signaling and HCC progression [91,97]. In addition to this, HCV-encoded protease NS3-4A could strengthen and prolong TGF-β-induced phosphorylation levels of Smad2/3 [98]. Tripartite motif containing 52 (TRIM52) is highly expressed in HCC tissues and promotes HCC cell proliferation and motility [159]. It was suggested to enhance TGF-β signaling by promoting proteasomal degradation of PPM1A [158]. Transcriptional intermediary factor 1 gamma (TIF1γ) is able to mono-ubiquitinates Smad4 and inhibits its oligomerization with R-Smads, therefore antagonizing TGF-β-mediated cytostatic effects or EMT [160]. Accordingly, decreased expression of TIF1γ in advanced HCC contributes to EMT, invasion and metastasis [160]. Interestingly, TGF-β induces intracellular release of Ca^2+^ ion in human hepatoma cells, by increasing the expression levels of Na^+^/Ca^2+^ exchanger 1 (NCX1) and the canonical transient receptor potential channel 6 (TRPC6), in addition to enhancing the NCX1-TRPC6 interaction. This contributes to TGF-β-mediated EMT, invasion and intrahepatic metastasis. Intriguingly, both NCX1 and TRPC6 seem to be required for TGF-β-induced R-Smads activation, creating a positive feedback loop [161].

## 7. Altering the Transcriptional Activity of Smads

TGF-β exerts various pathophysiological functions by activating both Smads and non-Smad signaling proteins [12,29,55]. On the one hand, these signaling molecules may directly exert their cellular functions, such as miRNA maturation processing executed by Smads and cytoskeletal rearrangement by small GTPases including RhoA, Rac1 and Cdc42 [37,52]. On the other hand, TGF-β signals would eventually lead to activation of downstream transcription factors including Smads and others, regulating target gene transcription [31,52,162]. Although exhibiting intrinsic DNA-binding ability and being required for TGF-β-induced both cytostatic effects and oncogenic functions, Smad proteins need to cooperate with other DNA-binding transcription factors to achieve highly efficient and selective DNA binding, yielding context- and cancer stage-dependent functions (Table 2 and Figure 5) [25,26,29]. In this regard, FoxO3 has been shown to interact with TGF-β-activated Smad2/3 in the nucleus, and mediates TGF-β-induced apoptosis of liver cancer cells [163,164]. Interestingly, this effect could be counteracted by casein kinase I-ε (CKI-ε) that phosphorylates FoxO3 at Thr32 [164]. AFP has been found reactivated in 70% to 85% of HCC cases and is widely used to stage aggressiveness and growth of liver cancer. However, the tumor suppressor p53 protein is able to anchor activated Smads on the AFP promoter, repressing AFP expression and HCC development [165]. Furthermore, p53 has been found to promote the tumor-suppressive functions of TGF-β by enhancing the expression of cell cycle- and apoptosis-related genes like p21, p15, Bim and DAPK, in collaboration with Smads [166]. Genetic loss of p53 could switch TGF-β from a tumor inhibitor to a promoter, by facilitating Snail expression and EMT [127,166,167]. Unlike the above, serum response factor (SRF) inhibits Smad binding to DNA and alleviates TGF-β-induced expression of p15 and p21 in HCC cells [168]. The HCV core protein could exert a similar function by interacting with the MH1 domain of Smad3, disrupting the Smad3/Sp1 complex formation and blocking their DNA binding. This leads to decreased expression of p21 and enhanced proliferation of HCC cells [99].

The transcriptional activity of TGF-β-activated Smad proteins is also subject to regulation by other nuclear proteins (Table 2 and Figure 5). We recently reported that the CXXC-type zinc finger domain-containing protein CXXC5 is transcriptionally upregulated by TGF-β/Smad signaling in HCC cells, and the CXXC5 protein in turn promotes TGF-β signaling by removing the histone deacetylase HDAC1 from activated Smad2/3, forming a novel positive feedback loop that plays a pivotal role in potentiating TGF-β-mediated growth inhibition of HCC cells [169]. Disruption of this regulatory loop by decreased CXXC5 expression as observed in most HCC tissues is then speculated to facilitate liver tumorigenesis [169,170]. Similarly, KLF17 is also required for TGF-β-mediated cytostatic effects in HCC. However, decreased expression of KLF17 in advanced HCCs renders cancer cells insensitive to TGF-β-induced tumor-inhibitory effects. KLF17 interacts with Smad3 and potentiates its DNA binding and transcriptional activity [171]. On the contrary, EVI acts as a transcriptional repressor for Smad3 via direct binding. It is upregulated in a subset of primary HCC and is associated with larger tumor size, blunting TGF-β-induced growth inhibition [172].

In advanced liver cancer, TGF-β-induced non-Smad pathways activate various transcription factors, which cooperate with Smads to induce the expression of certain sets of target genes and promote cancer development [10,12,25,26,29,55]. The cytosolic phospholipase A2α (cPLA2α), a rate-limiting enzyme in producing prostaglandin (PG), is soon activated upon TGF-β stimulation in both primary hepatocytes and human HCC cells, via ERK and p38 MAPK pathways [173]. cPLA2α then proceeds to activate PPARγ, which interacts with R-Smads and counteracts TGF-β-induced cytostasis [174]. Furthermore, activated cPLA2α may also potentiate TGF-β-mediated HCC malignancy by activating PI3K/Akt signaling [175]. In support of this, cPLA2α is highly expressed in metastatic HCC cell lines and at the invasive edge in HCC tissues [175]. Together, contextual expression and activation of transcription factors or transcriptional co-factors are critical to determine the genomic binding sites and transcriptional activity of TGF-β-activated Smads, giving rise to precise control of TGF-β target genes and functions (Table 2 and Figure 5).

## 8. Conclusions and Outlooks

Since the basic framework elucidation of TGF-β/Smad signaling almost two decades ago, understanding how TGF-β exerts its multi-faceted and even opposite pathophysiological functions, like those in cancer, has become a persistent pursuit [10,12,26,27,29,31]. Beyond all doubt, spatiotemporal regulation of TGF-β/Smad signaling plays a pivotal role in controlling its signaling robustness, duration, specificity and plasticity, and consequently the signaling readout [30,176,177]. Plenty of regulators and various mechanisms have been unveiled to operate on TGF-β signaling, from ligand bioavailability, receptor stability and activity, Smad signaling activity, to the contextual control of Smad transcriptional activity [34,35,36,37,41,162]. These regulators fine-tune TGF-β signaling to determine the paradoxical roles of TGF-β in liver cancer [9,10,12,154,166].

Evading TGF-β-mediated growth inhibition is a prerequisite for tumorigenesis in different organs [10,12,27]. In some cancer types including colon cancer, pancreatic cancer and gastric cancer, inactivating mutations and epigenetic silencing of TGF-β receptors or Smads are frequently observed. However, these genetic alterations are relatively rare in liver cancer [60,61]. On the contrary, signaling alterations have been found in liver cancer to alleviate the cytostatic branch of TGF-β actions. For instance, JNK-mediated linker region phosphorylation of R-Smads and altered Smad transcriptional activity play a crucial role [154].

In advanced liver cancer, TGF-β acquires a tumor-promoting ability as to cancer cell EMT and motility [10,12]. This is achieved by enhanced ligand bioavailability, altered expression and activity of TGF-β receptors, stabilization of activated Smad2/3 or their linker region phosphorylation, and guidance of Smads to activate expression of oncogenes. It is worth noting that TGF-β-induced activation of non-Smad pathways such as those mediated by PI3K/Akt, ERK/JNK MAPKs, β-catenin and NFκB, are closely involved in channeling, skewing and even switching the functions of TGF-β during liver cancer progression [10,29]. These signaling molecules may affect Smad signaling directly by protein-protein interaction or PTMs, or by regulating the transcriptional activity of Smad proteins via downstream transcription factors [30,52]. Furthermore, in addition to acting on cancer cells, TGF-β is also enriched in the tumor microenvironment, which could be transformed by TGF-β to facilitate liver malignant progression [10,178,179].

However, some important issues remain to be addressed. First, how do various regulatory mechanisms coordinately modulate the intensity, duration and plasticity of TGF-β signaling, thereby jointly determining the development of liver cancer? Second, there are still some gaps in understanding how different non-Smad pathways together exert their influences on Smad signaling and determine the functional readout of TGF-β. Third, how TGF-β function is switched from tumor-suppressive to oncogenic needs more in-depth investigations. Fourth, how can we translate these basic research results into clinical applications? Progress in understanding these questions would not only provide a more comprehensive picture of the role and mechanism of TGF-β in liver cancer, but also afford opportunities to target the TGF-β pathway in the therapeutic intervention of liver cancer.

## Figures and Tables

**Table 1:** Effects of HBV and HCV on TGF-β/Smad signaling.

Regulators	Functions	References
HBV	HBV X protein (HBx) could enhance TGF-β expression in liver and drive liver cancer progression.	[82]
	HBx promotes PPM1A degradation to enhance the tumor-promoting Smad signaling.	[91]
	HBx alters the phosphorylation pattern of Smad3, averting its activity from tumor-suppressive to oncogenic.	[92,93]
	Attenuates TGF-β-induced apoptosis in HCC cells by enhancing Smad7 expression.	[94]
HCV	HCV core protein increases TGF-β level in transgenic mice, by engaging thrombospondin-1.	[88]
	HCV core protein promotes HepG2 cell proliferation and chemoresistance by enhancing Smad7 expression and subsequently attenuating the cytostatic effects of TGF-β.	[95]
	Shifts the TβRI/pSmad3C/p21 tumor-suppressive pathway to the JNK/pSmad3L/c-Myc oncogenic signaling.	[92]
	Induces JNK-mediated linker phosphorylation of Smad2 and generates pSmad2L/C, contributing to HCC development.	[96]
	The HCV nonstructural protein 3 (NS3) promotes ubiquitination and degradation of PPM1A, thereby promoting Smad signaling and HCC progression.	[97]
	HCV-encoded protease NS3-4A strengthens and prolongs TGF-β-induced phosphorylation levels of Smad2/3.	[98]
	HCV core protein disrupts the Smad3/Sp1 complex and blocks their binding to the promoters of CDK inhibitors.	[99]

**Table 2:** Regulation of Smad Proteins in Liver Cancer.

Regulators	Functions	Alternation of the Regulators	References
FHL1	Exerts a tumor-suppressive role in liver cancer by triggering CK1δ-mediated and TGF-β receptor-independent phosphorylation of R-Smads.	Decreased in HCC tissues	[152]
NEK6	Exerts a tumor-promoting effect by interacting with Smad4 and attenuating the anti-proliferative effect of TGF-β.	Undetermined	[153]
IL-37	Inhibits Smad3-mediated oncogenic effects by inducing a conversion from the pSmad3L/c-Myc signaling to the pSmad3C/p21 signaling.	Decreased in human HCC tissues and cell lines	[157]
ELF	Acts as an adaptor protein for Smad3 and Smad4, therefore promoting the cytostatic effects of TGF-β in liver cancer. ELF^+/-^ mice spontaneously developed HCC.	Reduced in liver cancer tissues	[147,148,149]
PRAJA	Alleviates the tumor-inhibitory effect of TGF-β by associating with both ELF and Smad3 upon ligand stimulation.	Elevated in liver cancer tissues	[150]
GRK2	Interferes with Smad-mediated cytostatic effects by inhibiting TGF-β-induced C-terminal phosphorylation of Smad2/3.	Undetermined	[151]
TNF-α/ IL-1β	Promotes hepatocyte proliferation by inducing JNK-mediated linker phosphorylation of Smad3 and enhancing c-Myc expression.	Undetermined	[154]
MUC1	Converts the pSmad2/3C/p21 signaling to the pSmad2/3L/c-Myc oncogenic signaling by inducing JNK-mediated linker phosphorylation of Smad2/3.	Overexpressed in HCC cell lines	[83,84,156]
TRIM52	Promotes liver cancer progression by inducing PPM1A degradation and enhancing the pSmad2/3C levels.	Highly expressed in HCC tissues	[158,159]
TIF1γ	Antagonizes TGF-β-mediated cytostatic effects in the early stage and inhibiting EMT in the late stage by mono-ubiquitynating Smad4 and inhibiting its oligomerization with R-Smads.	Reduced in advanced HCCs	[160]
NCX1/ TRPC6	Be required for TGF-β-induced R-Smad activation and contribute to TGF-β-mediated EMT, invasion and intrahepatic metastasis.	Elevated in human HCC cells	[161]
CXXC5	Removes the histone deacetylase HDAC1 from activated Smad2/3 to potentiate TGF-β-mediated growth inhibition of HCC cells.	Decreased in HCC tissues	[169,170]
KLF17	Interacts with and enhances the transcriptional activity of Smad3, thereby facilitating TGF-β-mediated cytostatic effects in HCC.	Decreased in advanced HCCs	[171]
SRF	Attenuates the cytostatic functions of TGF-β by inhibiting Smad-DNA binding in HCC cells.	Undetermined	[168]
EVI	Acts as a transcriptional repressor for Smad3 to blunt TGF-β-mediated growth inhibition of HCC cells.	Elevated in a subset of primary HCCs	[172]
FoxO3	Interact with TGF-β-activated Smad2/3 and mediates TGF-β-induced apoptosis of liver cancer cells.	Undetermined	[163,164]
cPLA2α	Counteracts TGF-β-induced cytostasis by activating PPARγ and inhibiting R-Smad activity, and promotes HCC cell proliferation, EMT, migration and invasion by activating PI3K/Akt signaling.	Highly expressed in metastatic HCC cell lines and at the invasive edge in HCC tissues	[173,174]
p53	Cooperates with Smads to enhance the expression levels of cell cycle- and apoptosis-related genes like p21, p15, Bim and DAPK, and meanwhile to inhibit those of AFP and Snail.	Reduced or mutated in liver cancer	[127,165,166,167]

## Abbreviations

HCC:	hepatocellular carcinoma
TGF-β:	Transforming growth factor beta
BMP:	bone morphogenetic protein
GDF:	growth and differentiation factor
R-Smad:	receptor-regulated Smad protein
Co-Smad:	common Smad
I-Smad:	inhibitory Smad
pSmad3L:	the linker-phosphorylated form of Smad3
pSmad3C:	the C-terminus-phosphorylated form of Smad3
MAPK:	mitogen-activated protein kinase
ERK:	extracellular signal-regulated kinase
PTM:	post-translational modification
HBx:	hepatitis B virus X protein
HCV NS3:	HCV nonstructural protein 3
MMP:	matrix metalloproteinase
MEF2:	myocyte enhancer factors 2
Egr1:	Early growth response factor 1
HSPG:	heparan sulfate proteoglycan
HSC:	hepatic stellate cell
IQGAP:	IQ motif-containing GTPase activating protein
XIST:	X-inactive specific transcript
FHL:	four-and-a-half LIM protein
NEK6:	NIMA-related kinases 6
NCX1:	Na^+^/Ca^2+^ exchanger 1
TRPC6:	transient receptor potential channel 6
SRF:	serum response factor
cPLA2α:	cytosolic phospholipase A2α
